# The morphological and chemical properties of fine roots respond to nitrogen addition in a temperate Schrenk’s spruce (*Picea schrenkiana*) forest

**DOI:** 10.1038/s41598-021-83151-x

**Published:** 2021-02-15

**Authors:** Haiqiang Zhu, Jingjing Zhao, Lu Gong

**Affiliations:** 1 College of Resources and Environment Science, Xinjiang University, Urumqi, China; 2 Ministry of Education, Key Laboratory of Oasis Ecology, Urumqi, China; 3 School of Life Sciences, Sun Yat-Senen University, Guangzhou, China

**Keywords:** Ecology, Forest ecology

## Abstract

Fine roots (< 2 mm in diameter) play an important role in belowground ecosystem processes, and their physiological ecology is easily altered by nitrogen deposition. To better understand the response of physiological and ecological processes of fine roots to nitrogen deposition, a manipulation experiment was conducted to investigate the effects of exogenous nitrogen addition (control (0 kg ha^−1^ a^−1^), low (5 kg ha^−1^ a^−1^), moderate (10 kg ha^−1^ a^−1^), and high nitrogen (20 kg ha^−1^ a^−1^)) on the biomass, morphological characteristics, chemical elements and nonstructural carbohydrates of fine roots in a *Picea schrenkiana* forest. We found that most fine roots were located in the 0–20 cm of soil layer across all nitrogen treatment groups (42.81–52.09% of the total biomass). Compared with the control, the biomass, specific root length and specific root area of the fine roots increased in the medium nitrogen treatment, whereas the fine roots biomass was lower in the high nitrogen treatment than in the other treatments. In fine roots, nitrogen addition promotes the absorption of nitrogen and phosphorus and their stoichiometric ratio, while reducing the content of nonstructural carbohydrates. The content of nonstructural carbohydrates in the small-diameter roots (< 1 mm in diamter) in each nitrogen treatment group was lower than that in the large-diameter roots. Correlation analysis showed that soil carbon and nitrogen were positively correlated with fine root biomass and specific root length and negatively correlated with the nonstructural carbohydrates. Our findings demonstrate that medium nitrogen addition is conducive to the development of fine root morphology, while excessive nitrogen can suppress the growth of root systems.

## Introduction

Fine roots (< 2 mm in diameter) are the main organ through which plants absorb nutrients and water; therefore, these structures play a key role in carbon (C) distribution and nutrient cycling of terrestrial ecosystems^1,2^. Fine roots are the most active part of the root system and are sensitive to variations in soil and atmospheric environments due to nitrogen (N) deposition^3^. Nitrogen is not only an essential element for plant growth but also a limiting factor for tree growth and productivity in temperate forests^4^. Nitrogen deposition can affect forest productivity (e.g., plant biomass), soil nutrient cycling (e.g., soil N availability) and tree root systems (e.g., root production and morphology)^5,6^. Fine roots can adapt to changes in soil nutrient availability and the environment through changes in the plasticity of nutrient capture^7^. Therefore, exploring fine root responses to N deposition is necessary to understand the overall response of belowground ecosystem processes to environmental changes.

Over the past century, human activities such as rapid industrialization, the burning of fossil fuels, and the use of N fertilizers have led to a 3–5-fold increase in atmospheric N deposition^8^, which may affect soil nutrient availability and fine root growth^9^. Several traits of root morphology can reflect the ability of trees to obtain available nutrients in soil^10^. For instance, the specific root length (SRL) and specific surface area (SRA) of fine roots are important parameters for measuring fine root resource allocation and nutrient absorption^11^. Fine root tissue density (RTD) has been used as an important indicator of plant survival and growth strategy^12^. Extensive studies have shown that fine root morphology is closely related to the changes in soil nutrient availability caused by N deposition, although the degree of many responses is still controversial^13,14^. For example, in N-deficient forest ecosystems, nitrogen addition can increase the SRL of fine roots, which allows the roots to obtain more nutrient and water resources^15^. However, excessive N deposition may reduce fine root SRL and the ability to acquire nutrients^10,16^. Other studies have suggested that the N addition has no significant effect on SRL^17^. Additionally, different responses have been observed between soil N availability and RTD and SRL. For example, Ostonen et al.^10^ found that the RTD and SRL of Norway spruce decreased with increasing soil N availability, but Comas and Eissenstat^18^ found that RTD was significantly negatively correlated with SRL in temperate forests. There are three different types of responses of fine root biomass to N deposition: increased, unchanged or decreased^17,19^. The discrepancies in the responses of fine root morphology to N deposition may be ascribed to plant species (such as carbon allocation strategies), habitat conditions, and soil environments. Thus, studying the responses of fine root biomass and morphology to N deposition is necessary for understanding the impact of N deposition on the belowground processes.

Elucidating the chemical components of fine roots are necessary for understanding changes in their external morphology and function. Nitrogen and phosphorus (P) are often limiting macronutrient elements, and their dynamic changes can reflect the growth rate and nutrient limitation of plants. In N-limited temperate forest ecosystems, the fine root N content increased with increasing soil N and P contents due to N addition^17,20,21^. However, the contents of N and P in fine roots decreased with greater N availability in other studies^22^. In forest ecosystems, a change in N and P can further lead to a transformation from the limitation of a single nutrient resource to the limitation of both N and P^22,23^. This may be in part due to differences in the nutrient absorption and utilization of roots of different sizes. Nonstructural carbohydrates (NSC) are related to fine root respiration and can provide energy for new root growth and nutrient absorption. Changes in the content of NSC affect plant response strategies to environmental changes^24^. Moderate N addition may increase NSC in fine root and promote the absorption of N^25^, while excessive N can reduce the content of NSC. To date, research efforts have focused on the physiological responses of fine roots to N deposition, but the biological and abiotic correlates of fine root dynamics have received less attention due to the genetic basis of these traits for most tree species, the complexity of fine root structure, and the differences in soil environment and research methods used^9,17^. Therefore, research on the response of fine roots to N deposition in different regions and species is urgently needed.

The branched structure of fine roots may influence their functions, e.g., their roles in plant chemistry, morphology and physiology^26,27^. The responses of fine roots to N deposition differs between diameter classes. For example, the contents of N and P in fine roots was shown to decrease with increasing root order, while the NSC content showed the opposite pattern^28^. Some studies have found that the effects of N deposition are greater on lower order roots than on higher order roots^9,29^. Other studies have shown that the biomass of fine roots in the upper soil layers is significantly higher than that in other soil layers^15^ and that N addition significantly increases the SRL and SRA of fine roots in the upper soil layer (0–30 cm)^30^. However, Yan et al.^31^ demonstrated that N addition reduced the SRA of fine roots. The vertical distribution of fine roots and their response to N addition not only determine the strategies plants use to obtain soil resources but also reflect the adaptability of plants to the environment. Knowing how the morphological and chemical properties of fine roots in different diameter classes and soil layers change with N deposition is helpful for understanding the mechanisms of plant adaptation strategies to environmental changes in arid zones.

Tianshan Mountain is an important part of the arid mountain-basin system and contains the largest mountain forest distribution area (N-deficient ecosystem) in Xinjiang. Schrenk’s spruce is the dominant species in the Tianshan forest ecosystem and plays an important role in fixing N, releasing oxygen, regulating the climate, and maintaining the ecological environment. However, the N deposition in this area has increased due to human activities, such as grazing, tourism and coal mining, which in turn affect the soil properties and belowground processes of Schrenk’s spruce forest. Fine roots are environmentally sensitive components of the belowground processes of forest ecosystems, and changes in their characteristics can reflect the ability of trees to acquire nutrients^10^. However, research on the influence of N deposition on the fine root characteristics of Schrenk’s spruce is still insufficient. We conducted an exogenous N addition experiment to explore the responses of the morphological and chemical properties of fine roots and their relationships with soil factors in different soil layers and diameter classes of Schrenk’s spruce to improve our understanding of N deposition in forest systems of arid areas. Here, we verify the following hypotheses: (1) medium N addition increases fine root biomass, SRL and SRA and decreases NSC content and (2) there are positive correlations between fine root physiological and ecological characteristics and soil N.

## Results

### Variations in fine root biomass

**Figure 1:**
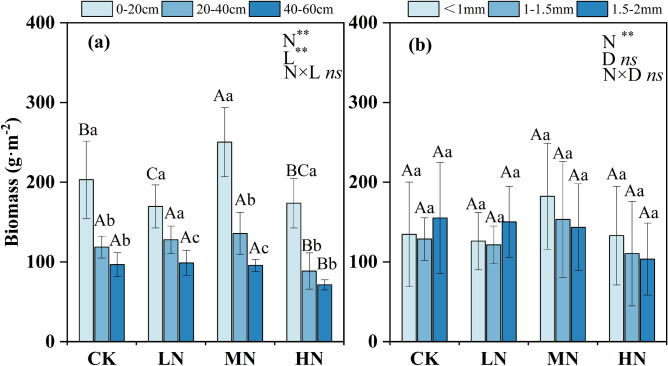
Fine root biomass for N treatments of different (**a**) soil layers and (**b**) root diameter classes. Lowercase letters indicate difference between soil layers (**a**) and between diameter classes (**b**); capital letters indicate differences between N treatments in the same soil layer (**a**) and diameter class (**b**). *CK* control, *LN* low N treatment, *MN* medium N treatment, *HN* high N treatment. The error bars represent the standard deviation. *N* nitrogen level, *L* soil layers, *D* diameter class. **P < 0.01 level of significance; *P < 0.05 level of significance; *ns* no significant.

The multivariate analysis of variance indicated that the N treatment had a significant effect on fine root biomass (Fig. 1, Table S1). The fine root biomass was significantly higher in the 0–20 cm layer than in the other soil layers in each treatment (*P* < 0.05). In the four N treatment levels, the biomass in the 0–20 cm soil layer accounted for 42–52% of the total biomass (Fig. 1a). In the high nitrogen (HN) treatment, the fine roots biomass in the 0–20 cm, 20–40 cm and 40–60 cm soil layers decreased by 14%, 25%, and 26%, respectively, compared with that in the control (CK) (Fig. 1a). However, the fine root biomass showed a slight increase in the medium nitrogen (MN) addition treatment (Fig. 1a). No significant difference was observed in fine root biomass between the N treatments and diameter classes (Fig. 1b). In the CK and low nitrogen (LN) treatments, the biomass of fine roots 1.5–2 mm in diameter was higher than that of fine roots in other diameter classes; the fine roots in the 1.5–2 mm class accounted for 37% of the total fine root biomass, but no statistical significance was found (Fig. 1b). However, in the MN treatment, the fine root biomass of roots < 1 mm increased by 35.52% compared with than in the CK treatment (Fig. 1b).

### Variations in fine root morphology

**Figure 2:**
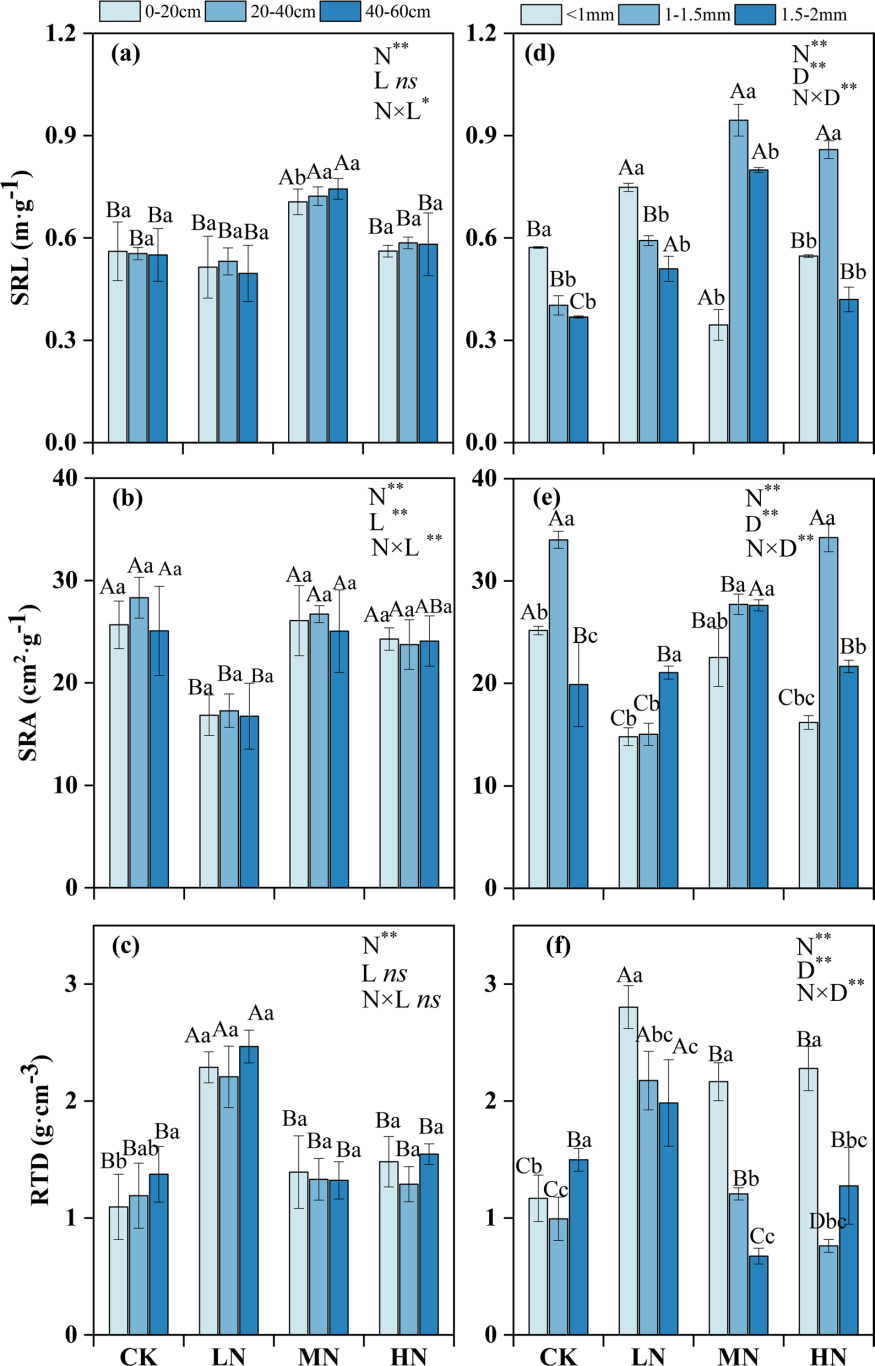
Mean fine root SRL, SRA, and RTD at different soil depths (**a**–**c**, respectively) and within different root diameter classes (**d**–**f**, respectively). *SRL* specific root length, *SRA* specific root area, *RTD* root tissue density. Lowercase letters indicate the differences between soil layers (**a**–**c**) or between diameter classes (**d**–**f**) in the same N treatment. Capital letters indicate the differences between N treatments within the same soil layer (**a**–**c**) or diameter class (**d**–**f**). *CK* control, *LN* low N treatment, *MN* medium N treatment, *HN* high N treatment. The error bars represent the standard deviation. *N* nitrogen level, *L* soil layers, *D* diameter class. **P < 0.01 level of significance; *P < 0.05 level of significance; *ns* no significant.

Nitrogen addition had a significant effect on SRL, SRA, and RTD (*P* < 0.01) (Fig. 2). Of the N treatments, the MN treatment significantly increased the SRL of each soil layer (*P* < 0.05) (Fig. 2a). The SRA value in the LN treatment was significantly lower than that in the other N addition treatments (*P* < 0.05) (Fig. 2b). The maximum value of RTD of the fine roots occurred in the LN treatment (2.20–2.46 g/cm^3^), which was 1.6–1.9 times higher than that in the other treatments (1.09–1.54 g/cm^3^) (*P* < 0.05) (Fig. 2c).

The interaction of N addition and diameter had a significant effect on SRL, SRA, and RTD (*P* < 0.01) (Fig. 2d–f). In the CK and LN treatments, the SRL of roots < 1 mm in diameter was significantly greater than that of roots in the other two diameter classes, and in the MN and HN treatments, the SRL of roots 1–1.5 mm in diameter was significantly higher than that of roots in the other two diameter classes (*P* < 0.05). Except in the LN treatment, the SRA of roots 1–1.5 mm in diameter was larger than that of roots < 1 mm and 1.5–2 mm in diameter (Fig. 2e). The RTD of was significantly higher in the roots < 1 mm in diameter in the N addition treatment than in the roots in the other two diameter classes (*P* < 0.05).

### Variations in the chemical properties of the fine roots

**Figure 3:**
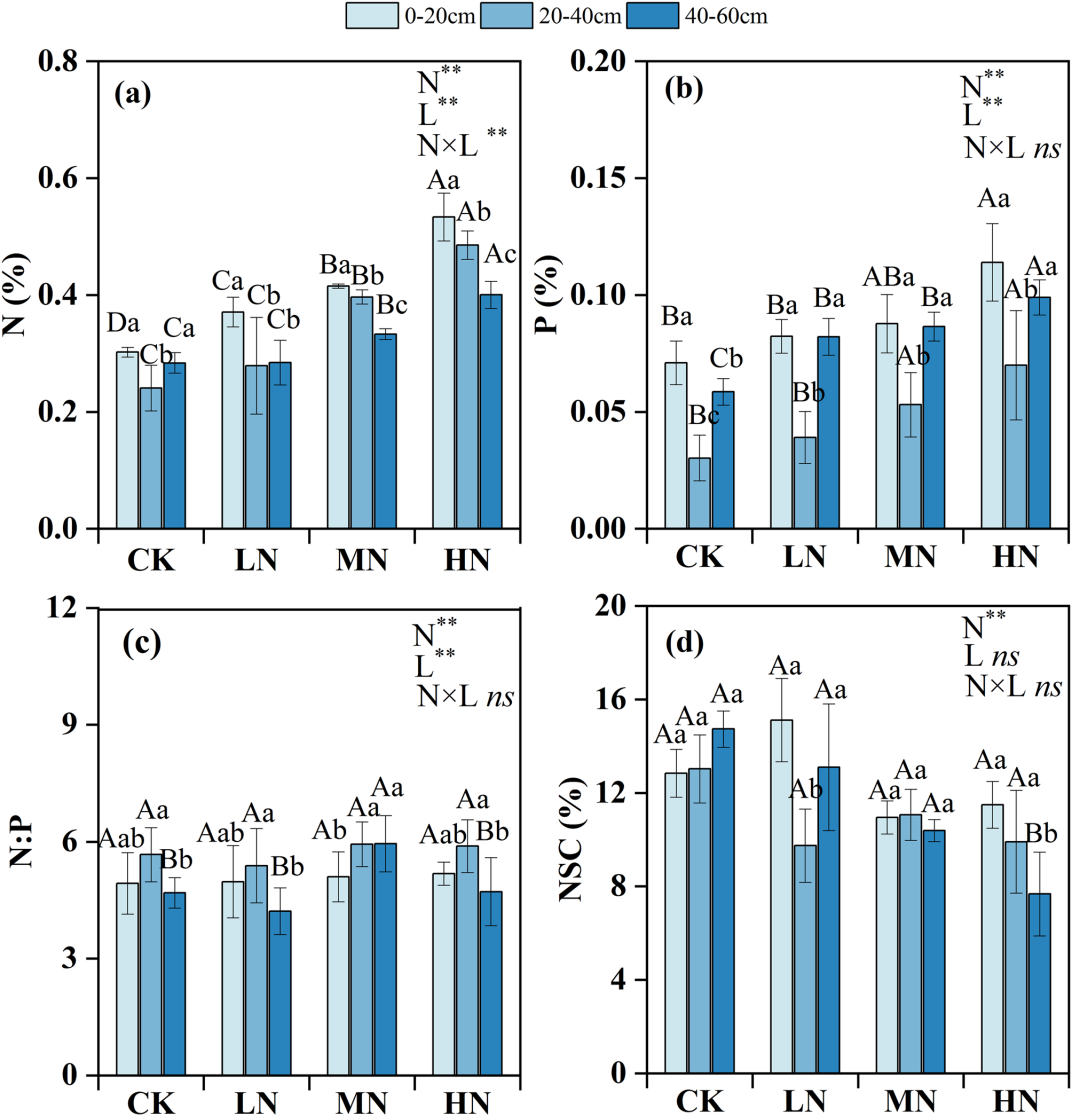
Mean fine root N content (**a**), P content (**b**), N:P (**c**), and NSC content (**d**) in different soil layers. *N* nitrogen, *P* phosphorus, *N:P* nitrogen to phosphorus, *NSC* non-structural carbohydrates. Lowercase letters indicate differences between soil layers in the same treatment; capital letters indicate differences between treatments within the same soil layer. *CK* control, *LN* N treatment, *MN* medium N treatment, *HN* high N treatment. The error bars represent the standard deviation. *N* nitrogen level, *L* soil layers. **P < 0.01 level of significance; *P < 0.05 level of significance; *ns* no significant.

The chemical properties of the fine roots differed significantly in response to N addition (*P* < 0.05) (Fig. 3). The fine root N and P contents (%) of each soil layer increased with increasing N addition (Fig. 3a,b). However, the NSC content (%) of the fine roots showed the opposite pattern (Fig. 3d). No significant differences in the fine root N: P were observed among the N addition treatments (Fig. 3c).

**Figure 4:**
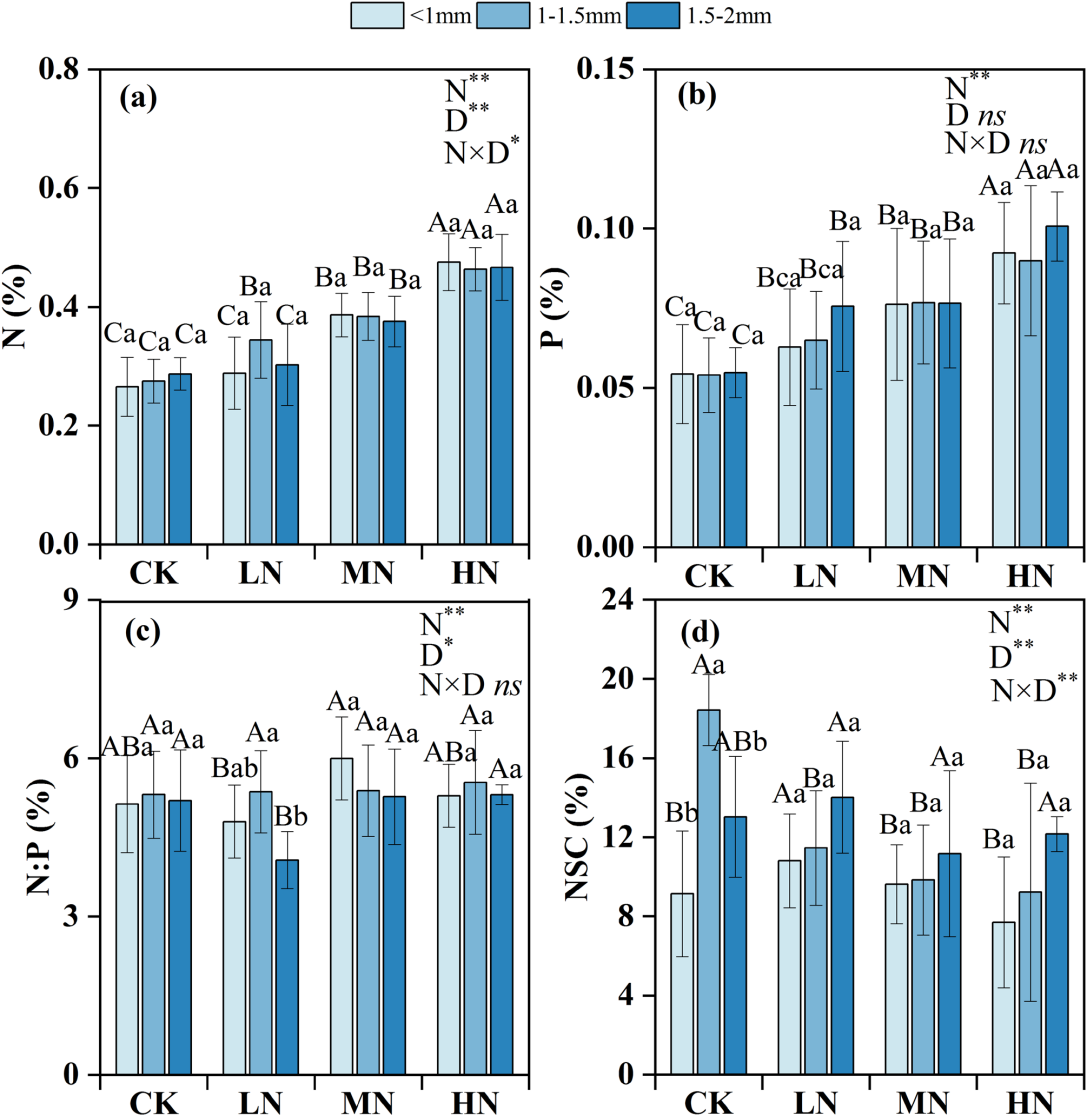
Mean fine root N content (**a**), P content (**b**), N:P (**c**), and NSC content (**d**) for different root diameter classes. *N* nitrogen, *P* phosphorus, *N:P* nitrogen to phosphorus, *NSC* non-structural carbohydrates. Lowercase letters indicate differences between diameter classes in the same treatment; capital letters indicate differences between treatments within the same diameter class. *CK* control, *LN* low N treatment, *MN* medium N treatment, *HN* high N treatment. The error bars represent the standard deviation. *N* nitrogen level, *D* diameter class. **P < 0.01 level of significance; *P < 0.05 level of significance; *ns* no significant.

There was a significant effect of N addition and diameter class on the fine root N, N:P, and NSC (Fig. 4). The maximum values of N and P in all the diameter classes occurred in the HN treatment; these values were significantly higher than those in all the other treatments (Fig. 4a,b). The minimum value of NSC appeared in the HN treatment, and no significant differences were found for this trait among the N addition treatments (Fig. 4d). Among all the treatment groups, the NSC contents were lower in the roots < 1 mm in diameter than in the roots in the other two diameter classes, but significance was found in only the CK treatment (Fig. 4d).

### Relationships between the fine roots characteristics and soil factors

**Figure 5:**
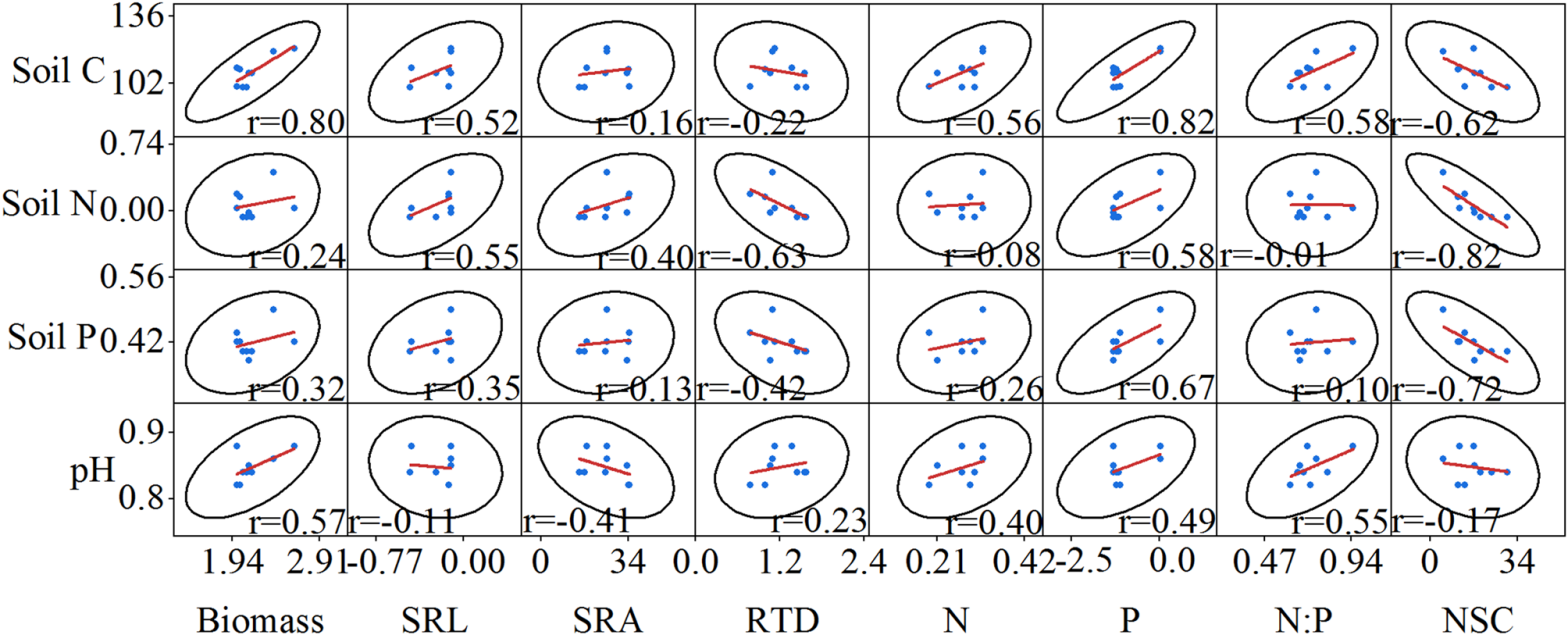
Pair-wise scatterplots between fine root characteristics and soil physical and chemical factors in the control (CK) treatment (n = 9). *Soil C* soil carbon, *pH* soil pH, *Soil P* soil phosphorus, *Soil N* soil nitrogen, *N:P* nitrogen to phosphorus, *Biomass* fine root biomass, *N* fine root nitrogen, *P* fine root phosphorus, *SRL* specific root length, *SRA* specific root area, *RTD* root tissue density, *NSC* non-structural carbohydrates.

**Figure 6:**
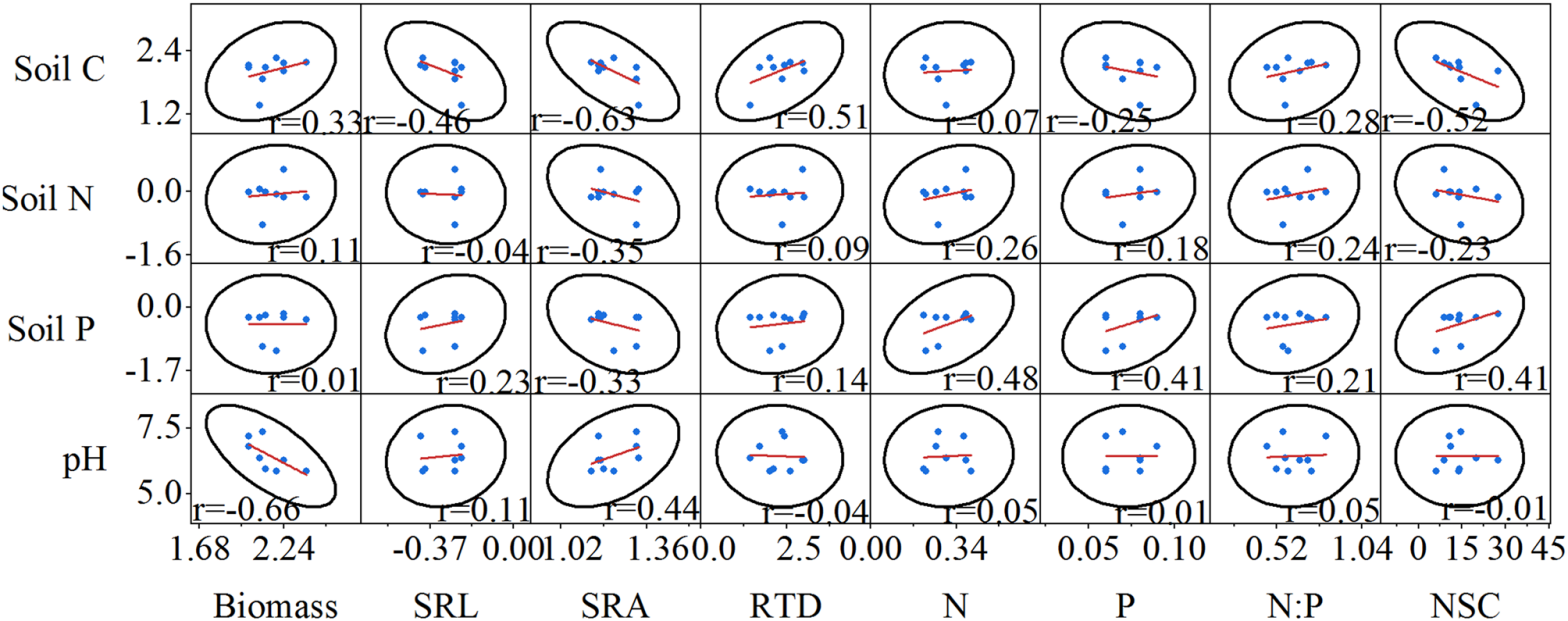
Pair-wise scatterplots between fine root characteristics and soil physical and chemical factors in the low nitrogen (LN) treatment (n = 9). *Soil C* soil carbon, *pH* soil pH, *Soil P* soil phosphorus, *Soil N* soil nitrogen, *N:P* nitrogen to phosphorus, *Biomass* fine root biomass, *N* fine root nitrogen, *P* fine root phosphorus, *SRL* specific root length, *SRA* specific root area, *RTD* root tissue density, *NSC* non-structural carbohydrates.

**Figure 7:**
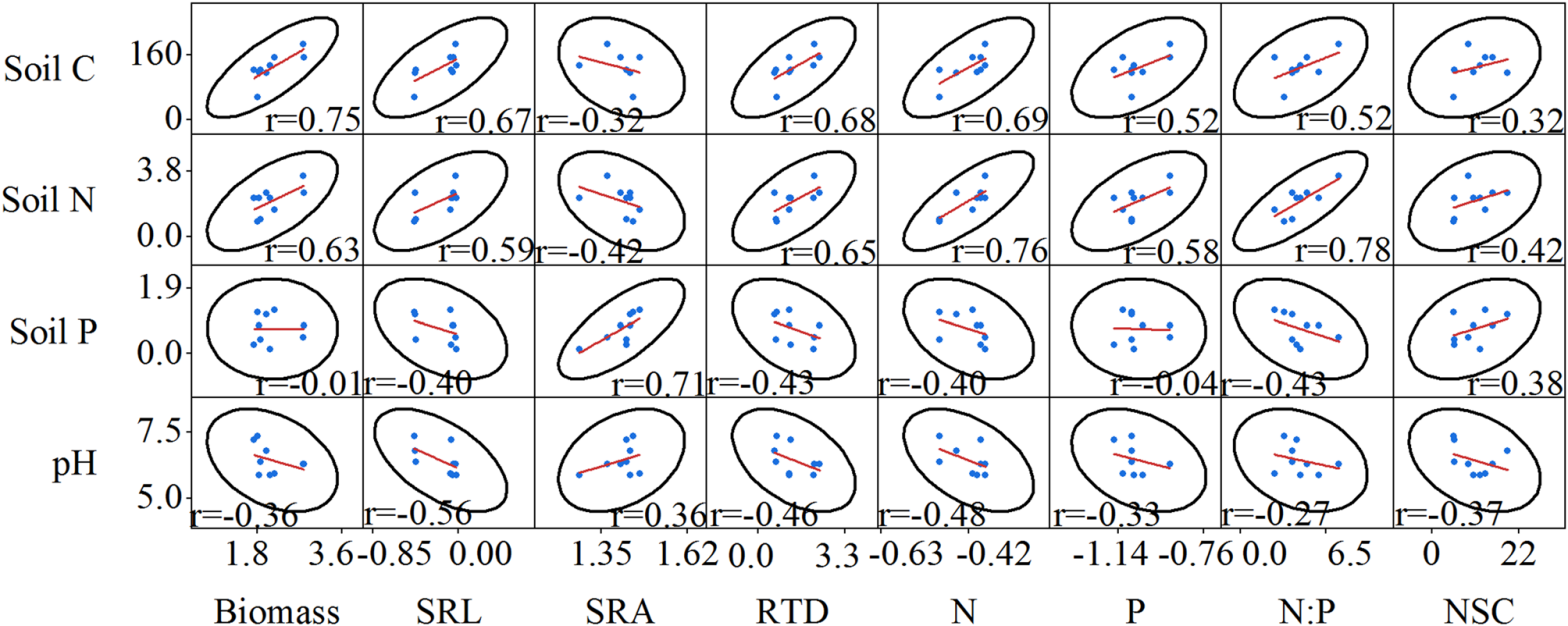
Pair-wise scatterplots between fine root characteristics and soil physical and chemical factors in the medium nitrogen (MN) treatment (n = 9). *Soil C* soil carbon, *pH* soil pH, *Soil P* soil phosphorus, *Soil N* soil nitrogen, *N:P* nitrogen to phosphorus, *Biomass* fine root biomass, *N* fine root nitrogen, *P* fine root phosphorus, *SRL* specific root length, *SRA* specific root area, *RTD* root tissue density, *NSC* non-structural carbohydrates.

**Figure 8:**
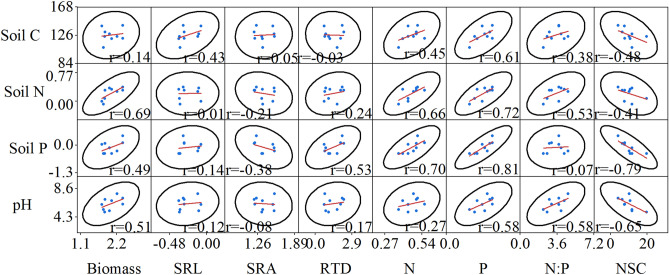
Pair-wise scatterplots between fine root characteristics and soil physical and chemical factors in the high nitrogen (HN) treatment (n = 9). *Soil C* soil carbon, *pH* soil pH, *Soil P* soil phosphorus, *Soil N* soil nitrogen, *N:P* nitrogen to phosphorus, *Biomass* fine root biomass, *N* fine root nitrogen, *P* fine root phosphorus, *SRL* specific root length, *SRA* specific root area, *RTD* root tissue density, *NSC* non-structural carbohydrates.

There were differences in the correlations between the soil physical and chemical factors and fine root morphology and chemical properties under the different N treatments. In the CK treatment, the soil C, N, and P were significantly positively correlated with the fine root SRL and significantly negatively correlated with NSC content (Fig. 5). A significant positive correlation was also found between the fine root biomass and soil C. The RTD was significantly negatively correlated with the soil N and P (Fig. 5). In the LN treatment, the soil C was positively correlated with the fine root biomass and N:P ratio (Fig. 6). In the MN treatment, the soil C and N were significantly positively correlated with the fine root biomass, SRL, N and N:P ratio (Fig. 7). In the HN treatment, the soil N and P were significantly positively correlated with the fine root N and P and negatively correlated with the NSC content (Fig. 8).

## Discussion

### Effects of N addition on fine root biomass and morphological characteristics

Nitrogen deposition can affect fine root biomass by changing soil nutrient availability and plant nutrient strategies^32^. Our findings indicated that the biomass of fine roots of Schrenk’s spruce was highest under the MN treatment in the 0–20 cm soil layer. In N-deficient ecosystems, moderate N addition is enhancing soil N availability and promotes plant growth, which in turn increases fine root biomass^33^. Moreover, the increased soil N is absorbed and utilized by plants, and the N in the soil does not have adverse effects on the root system due to excessive N accumulation^34^. Our results also revealed that the HN treatment decreased fine root biomass. First, nitrogen addition increased aboveground growth as a larger proportion of assimilated C can be used for aboveground growth when there is excess N in the soil. This leads to less C translocated belowground, reducing fine root growth and biomass^35^. Second, excessive N addition also leads to soil acidification and activation of aluminum ions in the soil^36^. Aluminum ions can bind to the cell walls and nucleic acids of fine roots and interfere with the energy exchange between fine roots and soils, thereby inhibiting the growth of fine roots^37^. Furthermore, the soil pH value was lower in the HN treatment than in the CK after a year of N addition (Fig. 1), and plant tried to avoid stress from acidification by investing less in root biomass. These results are inconsistent with observations of *Pinus koraiensis* and *Fraxinus mandshurica* forests, in which N addition increased fine root biomass^38^, and indicate that the response of fine roots to N deposition is controlled by the N addition dose, soil fertility and tree genetic characteristics. In addition, the biomass of fine roots < 1 mm in diameter in the MN treatment increased, but the difference was not statistically significant. First, fine roots < 1 mm in diameter are the main organs used for nutrient absorption and are more sensitive to the external environment^22^. Nitrogen addition may increase the branching of fine roots, which promotes the proliferation of small-diameter roots^15^. Moreover, the thinning of root diameter caused by N addition is conducive to the absorption and utilization of nutrients^15,21^. Our results showed that the fine root biomass was greatest in the 0–20 cm soil layer. Upper soil layers have greater available nutrients and better hydrothermal conditions and the soil texture in these layers is conducive to fine root growth and nutrient absorption^39^. However, the deeper soil layers contain leached nutrients (Fig. 1), which suppress the growth of fine roots^30^. Furthermore, surface soil layers are affected first when N is added, the foraging response of fine roots then leads to a change the fine roots in surface layer, which mediates the vertical fine root distribution^40^.

The SRL and SRA reflect the ability of fine roots to obtain nutrients and their competitiveness^41^. It is believed that changes in root morphology are related to physiological demands for N and cost–benefit balance^42^. We found that the MN treatment significantly increased the SRL of each soil layer, in agreement with previous studies showing that SRL and SRA increased by 105.4% and 24.9%, respectively, under comparable N treatments^9^. This indicated that the physiological activity and nutrient uptake rate of fine roots were increased by medium N addition. Increases in SRL may occur when the increase in soil N concentration fails to meet the physiological demands of Schrenk’s spruce, as greater SRL enhance nutrient foraging^11,15^. This conclusion is consistent with the positive correlation between the SRL of the fine roots and the soil N content. However, our results are inconsistent with the meta-analysis of Li et al.^17^, which showed that N addition failed to change the morphological characteristics of roots. These differences may be attributed to the following factors. First, fine roots are sensitive to the change in soil N caused by N addition in N-deficient forests. Second, the range of N addition duration in the meta-analysis was 0.2–13.6 years, and the effects produced by N addition varied with the length of the experimental period. Finally, the number of studies in temperate forests included in the meta-analysis was limited^17^, which may affect the significance of the findings of fine root responses to N deposition^43^.

### Effects of N addition on fine root chemical properties

Nitrogen deposition affects root metabolism and nutrient absorption by changing the chemical composition of fine roots^44^. We observed that N addition increased the contents of N and P in fine roots. The reason for this finding is that N addition might increase the N content in the soil, fine root phosphatase activity and nitrate availability, which promote the absorption of N by fine roots. In addition, plants may allocate relatively little energy to absorb N after N addition but allocate more energy to absorb P elements in the soil^13^. Fine roots can also enhance phosphatase activity to maintain P uptake^45^. The N:P ratio of fine roots can be used to determine the status of nutrient limitation^46^, and it is generally accepted that N:P ratios < 14 indicate that N is limited, while N:P ratios > 16 indicate that P is limited^47^. We found that the range of fine root N:P ratios under different N treatment levels was between 4.21 and 5.994, which was far lower than the global fine root N:P ratio of 11.5^48^. This indicates that N is still limited in Schrenk’s spruce forest, even under experimental N deposition. However, it is necessary to further study the threshold range of the fine root N:P ratio to accurately determine the status of plant nutrient limitations.

Nonstructural carbohydrates are related to fine root respiration and can provide energy for new root growth and nutrient absorption. Our results revealed that N addition reduced the NSC contents of the fine roots of Schrenk’s spruce because N addition increased the respiration of fine roots and accelerated the consumption of NSC^49^. Excessive N addition causes the energy consumed by fine roots to absorb N to be greater than their carbon input, resulting in a decrease in NSC content^50^. Fine roots have a hierarchical architecture, and there are some differences in the chemical and ecological characteristics of the fine roots of different diameter classes. In this study, the NSC content of the fine roots < 1 mm in diameter was slightly less than that of the roots in the other two diameter classes; however, significant differences were found in only the CK treatment. This may be because fine roots < 1 mm in diameter are composed mainly of low-order roots, these roots, which have a faster respiration rate than larger roots, are the most active part of the root system, and their nutrient absorption and root respiratory processes consume large amounts of NSC^20,51^.

### Relationship between fine root characteristics and soil environmental factors

Change in fine root morphological characteristics in response to changes in soil nutrients may cause shifts in root competitive strategies^52^. We showed that fine root biomass was positively correlated with soil C and N. Spruce allocates relatively more C to fine roots in the process of obtaining N, and the growth of fine roots can promote more N uptake^35^. Furthermore, the number and turnover of roots affects the C and nutrient content of soil^53^. It has also been shown that the availability of soil nutrients changes and that plant roots may increase their ability to absorb nutrients by changing their morphology^17^. In this study, SRL was significantly positively correlated with soil C and N. This may be because the SRL of fine roots is closely related to nutrient acquisition in nutrient-limited soils^11^. Fine root elongation and branch proliferation augment the ability of Schrenk’s spruce to access nutrients and water^54^. Nonstructural carbohydrates not only provide C for plant growth and reproduction but also serve as the substrate of respiration and metabolism^55^. When root and soil N increases, fine roots need to consume a larger amount of NSC to maintain normal growth and increase root respiration^56^. We found a significant negative correlation between the RTD and SRL of fine roots, which may be because the increase in the SRL of fine roots is indicative of another synchronous response of fine roots, i.e., the decrease in tissue density reflects a survival strategy of fine roots adapting to the soil environment^22^.

## Conclusions

Fine roots can alter their morphology in response to the environmental changes caused by N addition. The response of the fine root characteristics of Schrenk’s spruce to external N addition varied with the level of N addition, soil layer, and diameter class. Moderate N addition increased the SRL and biomass of the fine roots in each soil layer, while excessive N input suppressed fine root growth. The N addition promoted the absorption of N and P by fine roots. These results indicated that N is a vital factor for fine root growth in Schrenk’s spruce forests. Overall, understanding the responses of fine root morphology and chemical properties to N addition facilitates improving the management of Schrenk’s spruce forests under changing global climatic conditions.

## Materials and methods

### Study site

This study was conducted in an area of Schrenk’s spruce (*Picea schrenkiana*) forest near the Nanshan observation station (87.18° E, 43.47° N) of the Xinjiang Observatory at an altitude of approximately 2080 m. The region has a temperate continental arid climate with distinct cold and warm seasons. The average annual temperature is 0–4 ℃, and the average annual precipitation is approximately 500 mm^57^, mostly occurring from May to September. The average value of N deposition in this area is 5.33 kg ha^−1^ a^−1^^58^. Schrenk’s spruce is a constructive species in the forest ecosystem on Tianshan Mountain. The stands are mostly a pure forest, with heights of approximately 16 m and canopy densities of 0.6–0.8. The understory plants consist of *Geranium rotundifolium*, *Alchemilla tianschanica, Aegopodium podagraria*, etc., and the soil is mainly taupe forest soil developed over calcium rock differentiation material, which is weakly acidic and has a thick humus layer.

### Experimental design

To investigate the effect of N addition on the morphological and chemical properties of Schrenk’s spruce fine roots, we established a field experiment with a randomized block design. Three 20 × 20 m representative plots with similar altitudes (1942 m), slopes (24–26°), aspects (1°), and tree ages (78 a) were established in a Schrenk’s spruce forest, with at least 10 m spacing between each plot. Each plot was divided into four 3 × 3 m subplots with a 1 m buffer between each subplot. Iron plates were inserted at a depth of 50 cm between the subplots to prevent the transfer of soil nutrients. Four N addition treatments (control (0 kg ha^−1^ a^−1^), low N (5 kg ha^−1^ a^−1^), moderate N (10 kg ha^−1^ a^−1^) and high N (20 kg ha^−1^ a^−1^), were applied randomly in the four subplots of each plot. Schrenk’s spruce mainly takes up ammonium N from the soil. Research has shown that urea (CH_4_N_2_O) is converted into ammonium N by urease^59^, therefore, N was applied in the form of urea. Nitrogen additions began in October 2017 and continued once every two months until August 2018. The urea required for each treatment level was dissolved in 500 mL of deionized water (equivalent to the annual rainfall), and the solution was sprayed evenly in the sample area with a portable sprayer.

### Root and soil sampling

Fine root samples were collected in October 2018. In each subplot, three points were randomly selected within 1–1.5 m from the trunk. Fine roots were collected with a soil drill with an internal diameter of 10 cm from three depths (0–20 cm, 20–40 cm and 40–60 cm). The fine roots collected from the three points (within the same N addition group and soil layer) were thoroughly mixed and placed in a numbered sample bag. All the samples (n = 36) were placed in a cold storage box (2–4 ℃) and immediately transported to a laboratory. The soil particles and residual debris on the surface of the fine roots were washed away with deionized water, and live roots were selected according to color and activity. The live roots were more resilient than the dead roots when bent. Using Vernier calipers, the roots were classified (0–1 mm, 1–1.5 mm and 1–2 mm) and then put into labeled plastic bags. All the fine root samples were divided into two parts: one part for the determination of fine root morphology and the other for the analysis of chemical properties. All the samples were stored in a refrigerator at 2–4 ℃ for subsequent analysis. The soil was sampled similarly: a soil drill was used to collect samples at three depths (0–20 cm, 20–40 cm and 40–60 cm), nonsoil material (e.g., plant roots and human debris) was removed, and the soil was brought back to the laboratory in plastic bags. The samples were air-dried and then screened for preservation to determine the soil physical and chemical properties.

### Root morphology measurements

All the fine root samples were scanned using an Epson digital scanner, and a WinRHIZO professional root analysis system (WinRHIZO Pro STD 1600+, Regent Inc., Canada) was used to measure root length, surface area, and volume. These measurements were then used to calculate SRL = root length/dry weight (m/g), SRA = surface area/dry weight (cm^2^/g), and RTD = dry weight/volume (g/cm^3^).

### Determination of fine root chemical properties

The fine root samples were oven-dried at 65 ℃ for 48–72 h to a constant weight and then pulverized with a ball mill. The fine root N content was measured using the Kjeldahl method^60^. To analyze the P content, the fine roots were digested with H_2_SO_4_–H_2_O_2_ and the molybdenum antimony colorimetric method was applied^61^. The fine root NSC were measured using the phenol–sulfuric acid method^62^.

### Data analysis

Statistical analyses were performed using SPSS 17.0 (SPSS, IBM, USA). Multivariate analysis of variance was used to analyze the effects of different N treatment levels, diameter classes and soil layers on the fine roots, and least significant difference (LSD) test was used to analyze the differences in data in each group. Pearson correlation analyses were applied to identify the relationships between the soil properties and fine root characteristics. Origin 9.0 (Origin Lab, Massachusetts, USA) was used to draw graphics^63^.

## Supplementary Information

Supplementary Information.

## Data Availability

The fundamental data supporting the conclusions of this article are available as additional files in the “Supplementary information S1”.
