# Supplementary material for: The morphological and chemical properties of fine roots respond to nitrogen addition in a temperate Schrenk’s spruce (Picea schrenkiana) forest
Source: Sci Rep. 2021 Feb 15;11:3839. doi: 10.1038/s41598-021-83151-x (PMC7884734; doi:10.1038/s41598-021-83151-x)
Supplement: Supplementary file 1 — Supplementary Information. [file 41598_2021_83151_MOESM1_ESM.zip › Supplementary Dataset/Table S1.docx]

| Source of variation | df | *F* value | | | | | | | |
| --- | --- | --- | --- | --- | --- | --- | --- | --- | --- |
|  |  | Fine root  biomass | SRL | SRA | RTD | N | P | N:P | NSC |
| N | 3 | 7.218^**^ | 254.441^**^ | 341.950^**^ | 181.513^**^ | 165.748^**^ | 45.103^**^ | 5.748^**^ | 6.431^**^ |
| L | 2 | 76.868^**^ | 1.648 *ns* | 12.565 ^**^ | 7.415 *ns* | 52.649^**^ | 53.283^**^ | 12.985^**^ | 2.753*ns* |
| D | 2 | 0.435 *ns* | 821.326^**^ | 516.881^**^ | 198.822^**^ | 1.319 ^**^ | 2.716 *ns* | 4.442 ^*^ | 8.879^**^ |
| N×L | 6 | 1.863 *ns* | 2.369 ^*^ | 5.714 ^**^ | 2.112 *ns* | 7.827^**^ | 1.338 *ns* | 2.385 *ns* | 2.196 *ns* |
| N×D | 6 | 1.131 *ns* | 355.932^**^ | 233.240^**^ | 47.748^**^ | 2.205 ^*^ | 0.703 *ns* | 3.153 *ns* | 4.025^**^ |
| N×L×D | 12 | 0.630 *ns* | 1.164 *ns* | 8.194^**^ | 2.006 *ns* | 2.013 *ns* | 0.785 *ns* | 1.320 *ns* | 1.111*ns* |

**Table S1.** ANOVA results of the effects of N addition treatment (N), soil layer (L) and fine root diameter (D) on fine root morphology and chemical properties.

SRL, specific root length; SRA, specific root area; RTD, root tissue density; N, fine root nitrogen; P, fine root phosphorus; N:P, nitrogen to phosphorus; NSC, non-structural carbohydrates. ^**^*P* < 0.01 level of significance; ^**^*P* < 0.05 level of significance; *ns*, no significant.
