# Supplementary figures and images for: The morphological and chemical properties of fine roots respond to nitrogen addition in a temperate Schrenk’s spruce (Picea schrenkiana) forest
Source: Sci Rep. 2021 Feb 15;11:3839. doi: 10.1038/s41598-021-83151-x (PMC7884734; doi:10.1038/s41598-021-83151-x)

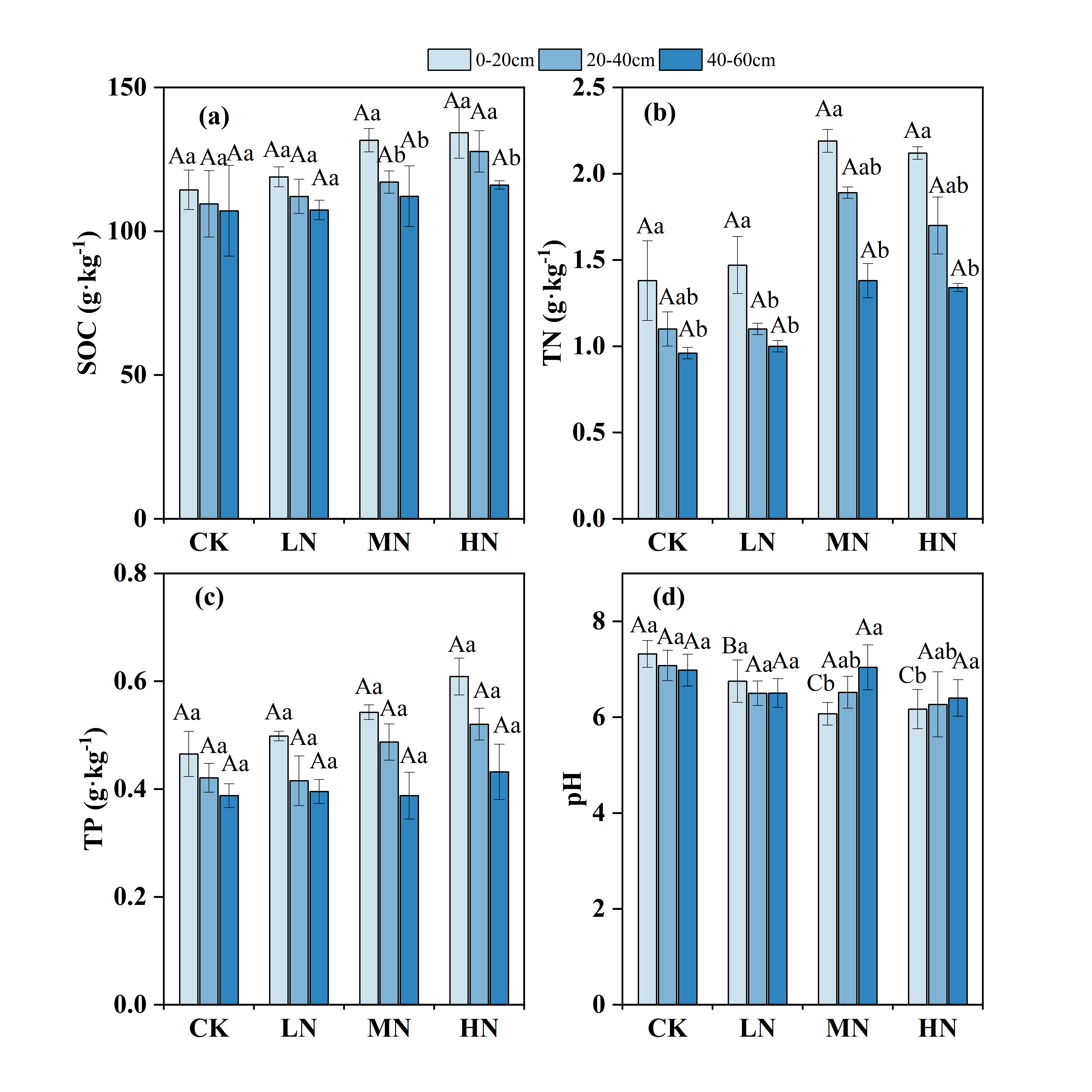

Supplement: Supplementary file 1 — Supplementary Information. [file 41598_2021_83151_MOESM1_ESM.zip › Supplementary Dataset/Figs1.tif]
